# Effect of biopsychosocial model of rehabilitation on functional disability and pain in patients after robotic-assisted lumbar fusion surgery

**DOI:** 10.1186/s13063-025-09129-6

**Published:** 2025-11-12

**Authors:** Jomi John, Vidyadhara S., Balamurugan T., Abhishek Soni, Madhava Pai Kanhangad, Karvannan Harikesavan

**Affiliations:** 1 Department of Physiotherapy, Manipal College of Health Professions Bengaluru, MAHE, Manipal, Karnataka India; 2 Manipal Comprehensive Spine Care Center, Manipal Institute of Robotic Spine Surgery, Manipal Hospitals, Bangalore, India; 3 Kasturba Medical College and Hospital Manipal, Udupi, India

**Keywords:** Biopsychosocial model, Robotic-assisted lumbar fusion, Lumbar fusion, Rehabilitation

## Abstract

**Background:**

The rate and prevalence of lumbar fusion surgeries are rapidly increasing, and these procedures effectively treat spinal disorders. Biological, psychological, and social factors play important roles in postoperative rehabilitation. Robotic-assisted surgeries reduce postoperative morbidities, and postoperative outcomes can be improved by interventions targeting biopsychosocial factors along with rehabilitation. Hence, this study aimed to determine the effect of biopsychosocial model of rehabilitation on pain in patients after robotic-assisted lumbar fusion surgery.

**Methods:**

An assessor-blinded randomized controlled trial will recruit 136 participants, and randomly allocate the participants into two groups. The intervention group will follow the biopsychosocial rehabilitation model, whereas the control group will be subjected to a routine standard protocol. Assessments will be done at baseline, 2, 4, and 12 weeks post-surgery. The normality of the data will be evaluated using the Shapiro‒Wilk test and analysis of variance with repeated measures or General linear models for intergroup differences. This protocol follows all required ethical guidelines.

**Discussion:**

This study will address the importance of the biological, psychological and sociological factors of rehabilitation. Analysis of the study results will provide insights into the importance of the biopsychosocial rehabilitation model of rehabilitation in patients after lumbar fusion.

**Trial registration:**

CTRI, CTRI/2024/12/077598. Registered on 03 December 2024.

**Supplementary Information:**

The online version contains supplementary material available at 10.1186/s13063-025-09129-6.

## Background

Lumbar fusion is an effective treatment option for a spectrum of spinal disorders, including traumatic, infectious, neoplastic, and degenerative pathologies of the lumbar spine [1]. It is widely used to treat lumbar spinal stenosis, degenerative spondylolisthesis, and isthmic spondylolisthesis [2]. The prevalence of lumbar fusion surgeries is rapidly increasing [3]. Recent studies reported that the rate of spinal fusion has increased by approximately 137% in recent decades [4]. Lumbar spine surgeries are associated with an increased rate of perioperative and postoperative morbidities, such as an extended duration of hospital stay, a need for prolonged rehabilitation, and psychological distress due to preoperative fear, pain, and disability before lumbar fusion surgery [5, 6].

There is a strong influence of pain intensity and psychological factors on variation in mental health, fear of movement/(re)injury, functional disability, and quality of life after lumbar fusion surgery [7]. These factors can negatively influence patient-reported outcomes after lumbar fusion. In recent decades, various surgical techniques have been introduced to minimize morbidities [1] of spine surgeries, such as minimally invasive surgery [8] and robotic-assisted spine surgery [9]. Robotic surgeries have emerged with 99% accuracy in pedicle screw fixation [10] and reduced re-surgery rates by 46% [11] and a fivefold reduction in complication rates [12]. A recent study that compared postoperative outcomes between robotic and open surgery revealed a significant reduction in the length of hospital stay and reduced exposure, but the study was limited in exploring patient outcomes following surgery [13].

The current biomedical model of rehabilitation after lumbar fusion surgery describes the physiological and anatomical causes of a patient’s illness. This approach is limited to considering the environmental, social, and psychological factors that cause patient illness [14]. Biological, psychological, and social factors can influence patients’ outcomes, such as pain intensity, functional activity, and quality of life. The biopsychosocial model of rehabilitation focuses on the integrated framework of patients’ biological, psychological, and social factors that influence disease and illness. Biopsychosocial approaches include behavioral treatment, cognitive behavior therapy, and coping skill training [15].

The biopsychosocial model has a positive impact on musculoskeletal conditions. Research investigating its application in postoperative management has revealed positive effects on catastrophizing pain management and functional outcomes [16]. However, a knowledge gap remains in assessing the impact of rehabilitation following robotic-assisted lumbar fusion. Currently, there is a lack of standardized rehabilitation programs tailored to advanced surgical techniques after lumbar fusion, and there is a paucity of knowledge on the impact of rehabilitation after robotic-assisted lumbar fusion [17]. Hence, this study aimed to determine the effect of biopsychosocial model rehabilitation on pain and function in patients after robotic lumbar fusion surgery.

### Objectives

The study’s primary objective is to find out the effect of biopsychosocial model of rehabilitation after robotic-assisted lumbar fusion surgery on functional disability using Oswestry Disability Index and pain using visual analogue scale.

The secondary objectives are as follows:

- To examine the effect of biopsychosocial model of rehabilitation after robotic-assisted lumbar fusion surgery on pain catastrophizing using Pain Catastrophizing Scale
- To evaluate the effect of biopsychosocial model of rehabilitation after robotic-assisted lumbar fusion surgery on quality of life using SF-36 questionnaire
- To find out the effect of biopsychosocial model of rehabilitation after robotic-assisted lumbar fusion surgery on functional mobility using Timed Up and Go test

## Methods

### Trial design

This is an assessor-blinded, randomized controlled trial with an interventional and control group.

### Study setting

Participants will be recruited from the Department of Spine Surgery, Manipal Comprehensive Spine Care Center, Manipal Hospitals Bangalore, on the basis of inclusion and exclusion criteria. The eligible and interested subjects attended a baseline assessment before the surgery, and informed consent was obtained from the subjects by the researcher.

### Eligibility criteria

The inclusion criterion for the present study was male and female patients aged 18–65 undergoing index single-level or two-level fusion surgery between the lumbar vertebrae L1 and S1. Eligible participants must be selected for lumbar fusion with decompression and have a primary diagnosis of conditions such as spinal stenosis, spondylosis, degenerative or isthmic spondylolisthesis, or degenerative disc disease.

The exclusion criteria for the study included patients diagnosed with inflammatory conditions such as rheumatoid arthritis and ankylosing spondylitis, as well as those with severe cardiorespiratory issues or neurological deficits classified as ASIA C or above. Individuals with severe psychiatric or psychological disorders, alcohol abuse, or those experiencing immediate postsurgical complications such as infections were also excluded. Additionally, patients with degenerative or idiopathic scoliosis, a history of malignancy in the spine, poor postoperative general health, spinal fractures, worsening existing symptoms, or the emergence of new symptoms will not be considered for participation.

### Withdrawal and drop-out criteria

The participants had the option to discontinue the study at any time or to exacerbate any serious adverse events. The subjects will be included in the analysis after completing the follow-up.

### Recruitment

Participants will be recruited from the Department of Spine Surgery, Manipal Comprehensive Spine Care Center, Manipal Hospitals Bangalore, on the basis of inclusion and exclusion criteria. The eligible and interested subjects attended a baseline assessment before the surgery, and informed consent was obtained from the subjects by the investigator.

### Assignment of interventions: allocation

**Fig. 1:**
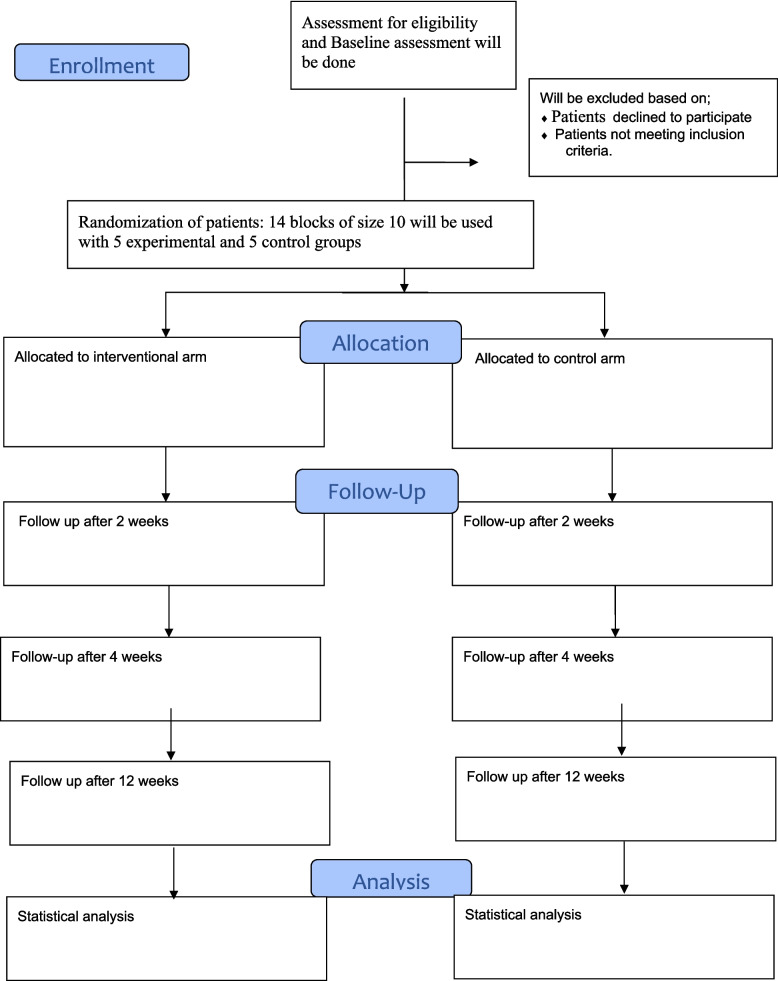
Flow of the study

The participants will be randomly allocated into the experimental and control groups using block randomization. Fourteen blocks of block size 10 will be used, and the allocation ratio is 1:1. Concealed allocation will be performed with nontransparent envelopes, which will in turn be signed by the respective supervisor, who is uninvolved in subject recruitment. Once the participant reports, one neutral arbitrator will pick an envelope and provide an intervention to the treating therapist. The study flow is demonstrated in (Fig. 1).

### Interventions

The patients were allocated to the interventional arm or the control arm. The control group interventions focused on treating the physiological and anatomical causes of the patient’s illness. Both groups will receive supervised treatment in IPD (two sessions), supervised treatment in OPD (six sessions), and unsupervised treatment from 4 to 12 weeks. Each session will last for approximately 40 to 45 min. Patients are instructed to perform the home exercises daily, and a self-reported diary will be used to evaluate patient compliance with the exercises and instructions.

### Biopsychosocial model of rehabilitation

The intervention group will participate in a rehabilitation program based on the biopsychosocial model of rehabilitation. The rehabilitation protocol is focused on the biological, psychological, and sociological factors influencing patients’ illnesses. This goal can be attained by treating physical symptoms, managing thoughts and cognitive processes, exercising, and providing family education. The biopsychosocial model of rehabilitation (Appendix: I) will include pain coping skill training, progressive muscle relaxation, pleasant activity, and education of family members along with exercise. The pain coping skill training and progressive muscle relaxation begin on first session which is postoperative day (POD) and advise patients to practice it when they experience an increase in pain. Components of biopsychosocial factors and exercise will be given throughout the sessions. On POD-1, the subjects will be instructed to use mini-practices during the day, and the education of family members will be provided. The participants will learn to identify and substitute automatic negative thoughts with constructive thoughts during the breach exercise and during the rest of the day. In later sessions, they learn how to alternate activities and rest, as well as how to manage negative thoughts. The participants are instructed to imagine calming pictures, such as the sea or the mountains, and to perform leisure activities such as reading. Each session will incorporate coping skills, which will be integrated into functional activities throughout the day [15, 18, 19]. As the final supervised session progresses, the biopsychosocial rehabilitation model will be addressed by the therapist in all of its parts. All the sessions except POD-1 ended with home advice and a demonstration of the home exercise program.

### Rehabilitation for the control group

The control group rehabilitation interventions (Appendix: II) will focus on treating the physiological and anatomical causes of the patient’s illness, including range of motion exercises, strengthening exercises, and advice for returning to normal activities. The rehabilitation begins with patient education and breathing exercises on POD-1. During POD-2 muscle activation exercise, range of motion exercises for the lower limb and walking with support are performed. The following sessions will consist of returning to normal activity advice, activating and strengthening exercises of muscles, and a demonstration of home exercises. Training intensity progression will be done on the basis of patients’ self-perception of pain.

### Outcomes

Baseline assessments will be performed before surgery using the Oswestry Disability Index, visual analog scale, Timed Up and Go test, Pain Catastrophizing Scale, and SF-36 questionnaire. The participants will be reexamined at 2, 4, and 12 weeks after surgery.

#### Primary outcomes

• Oswestry disability index
  It is presented as a score of 0–100, where the lowest score represents the minimal level of low back pain disability. The ODI has good test‒retest reliability, validity, and internal consistency in chronic back pain and spine surgery populations. The ODI has excellent telephonic reliability and validity (with a correlation coefficient of 0.98 and a range of 0.95–1). The minimum clinically important difference (MCID) has been reported to be between 4 and 10.5 points and is clinically significant [20].
• Visual analog scale (VAS)
  The VAS is a self-reported outcome measure used to assess the level of pain that a patient experiences. Pain is measured on an 11-point scale where 0 is considered no pain and 10 is the worst pain. The visual analog scale is a reliable and valid tool for measuring pain, with a minimum clinically important difference (MCID) of 1.4 cm (14% of the total length of the scale), which is clinically significant [21].

#### Secondary outcomes

**Fig. 2:**
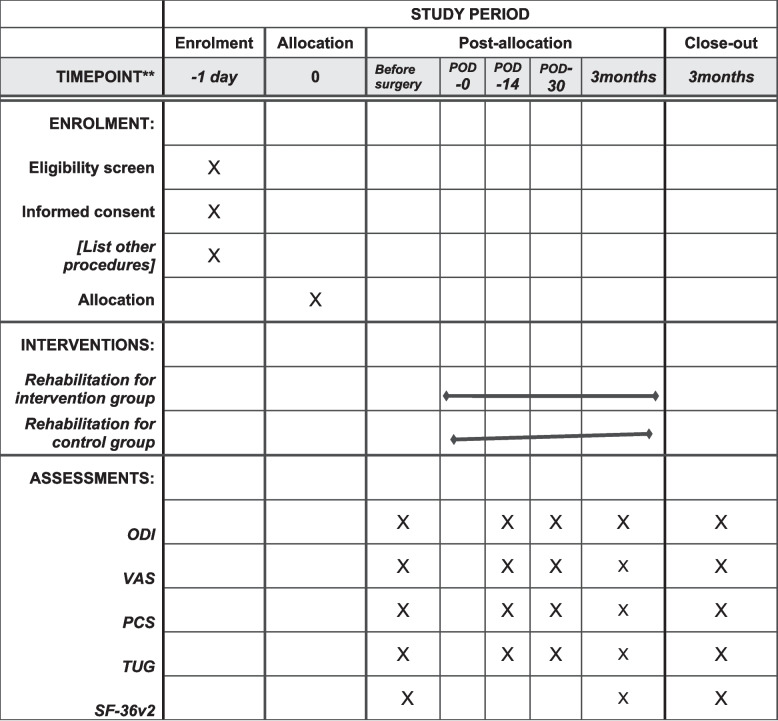
Time plan of the study

• Timed Up and Go test (TUG)
  In the Timed Up and Go test, the patient should get up from the chair, walk a distance of 3 m twice, and sit down as quickly as possible. A minimum clinically important difference (MCID) of 3.4 s was established, which is clinically significant [22].
• Pain catastrophizing scale (PCS)
  The PCS is a self-report questionnaire that measures an individual’s tendency to catastrophize pain. The PCS total scores range from 0 to 52. The PCS, as a full scale, is concluded to be a reliable measure with high test‒retest reliability [23].
• SF-36v2
  The SF-36v2 is a self-reported outcome measure. It consists of 36 questions and 8 subscales investigating physical function, pain, vitality, role Limitations, social functioning, mental Health, emotional problems, and general Health status. The scale is scored from 0 to 100, with 0 representing the lowest quality of Life and 100representing the highest quality of life [24, 25]. The questionnaire will be used for baseline assessment and final follow-up (Fig. 2).

### Sample size

The sample size was calculated using G*Power 3 and based on the Oswestry Disability Index standard deviation and effect size reported by Haoming Wang [26] et al. Study power is considered to obtain a *p*-value of less than 0.05. The power of the study is 0.80. Dropout will be considered up to 15% and calculated using the formula “n/1-dropout rate percentage”. The total number of participants in the study, including dropouts, is 136.

### Data analysis

The analysis of clinical outcomes will be performed using JAMOVI version 2.3.28. The normality of the data will be evaluated using the Shapiro‒Wilk test and analysis of variance with repeated measures or general linear models for intergroup differences. Only subjects completing all follow-ups will be included in the analysis.

The detailed analysis of the study results will provide valuable information on the significant influence of the biopsychosocial rehabilitation model on the overall functional disability, pain, and quality of life of patients who have undergone lumbar fusion surgery. Intention-to-treat analysis will be used to handle the missing values of participants who drop out from the study due to unavoidable reasons. MCMR test will be done, and “expectation maximization” test will be applied if the data is missing in random.

## Monitoring and data management

The institutional research committee and the Ethical committee of the Manipal hospitals has approved the current study. The data and information collected during the study will be securely stored in a protected research folder under the supervision of the corresponding author. The Doctoral Advisory Committee of Manipal Academy of Higher Education will monitor the study progression.

## Ethics

The study received ethical approval from the ethics committee of Manipal Hospitals Bangalore on 25th September 2024. The committee reviewed the study protocol, proforma, patient information sheet, and informed consent form.

## Discussion

Lumbar fusion is considered an effective treatment option for spinal disorders. In the last few decades, the prevalence of lumbar fusion surgery has increased, which has led to several new surgical strategies aimed at reducing postoperative complications. However, it is worth noting that biological, psychological, and social factors affect the outcomes of patients after lumbar surgery; however, current rehabilitation methods address only physiological and anatomical causes. However, the biological, psychological, and social factors of rehabilitation are likely needed to improve functional disability, pain, and other outcomes. This study aimed to determine the effect of the biopsychosocial model of rehabilitation after lumbar fusion.

The biopsychosocial model provides an integrated approach to the experience of pain, function, and mobility. Additionally, the model helps individuals manage their illness through self-management strategies. The biopsychosocial model of rehabilitation probably provides more beneficial effects than other conventional treatments do.

However, to the best of our knowledge, no studies have been conducted to investigate the effect of the biopsychosocial model of rehabilitation after lumbar fusion surgery, and there is not a single study involving robotic-assisted lumbar fusion surgery.

## Trial status

The present study is registered in the Clinical Trial Registry of India (CTRI/2024/12/077598, registered on 3rd December 2024). The study is currently in the recruitment stage. Recruitment began on 15th December 2024, and the estimated date of completion will be 14th April 2027.

## Supplementary Information

Additional file 1. Appendix I: Intervention for the experimental group. Appendix II: Standard rehabilitation protocol for the control group. Appendix III: informed consent form.

Additional file 2. SPIRIT checklist: Reporting checklist for protocol of a clinical trial.

## Data Availability

The data and information collected during the study will be securely stored in a protected research folder under the supervision of the corresponding author. Data will be shared in response to reasonable queries.
