# Supplementary material for: Effect of biopsychosocial model of rehabilitation on functional disability and pain in patients after robotic-assisted lumbar fusion surgery
Source: Trials. 2025 Nov 12;26:498. doi: 10.1186/s13063-025-09129-6 (PMC12613912; doi:10.1186/s13063-025-09129-6)
Supplement: Supplementary file 1 — Additional file 1. Appendix I: Intervention for the experimental group. Appendix II: Standard rehabilitation protocol for the control group. Appendix III: informed consent form. [file 13063_2025_9129_MOESM1_ESM.docx]

**APPENDIX: I**

Intervention for the experimental group

| **SL NO.** | **SESSION** | **INTERVENTION GROUP** |
| --- | --- | --- |
| 1 | SESSION 1  (POD 0) | - Overview of Biopsychosocial Model Rehabilitation - Introduction of Progressive Muscle Relaxation - Introduction of Pain coping skills - Early mobilization and beginning of rehabilitation |
| 2 | SESSION 2  (POD 1) | - Review progressive muscle relaxation and practices - Introduce “mini-practices” (brief relaxation) - Education of family members - DVT Prevention - Lumbopelvic stabilization exercises - Walking - Demonstration of home exercise program - (Dose: 3 sets X 10 reps daily) |
| 3 | SESSION 3  (POD 3) | - Review mini-practice and practices completed - Introduce activity/rest cycling - Spinal muscles strengthening exercises (Neutral spine control exercises) - Lumbopelvic stabilization (Maintaining neutral spine posture) - Walking |
| 4. | SESSION 4  (POD 5) | - Review activity/rest cycling and practices - Introduce concept of negative automatic thoughts - Spinal muscles strengthening exercise - Neutral spine control exercises - Walking |
| 5 | SESSION 5  (POD 7) | - Review coping thoughts and practices - Continue lesson on automatic thoughts - Introduce concept of coping thoughts - Strengthening exercise - Neutral spine control exercises - Walking - Home exercise program (Dose: 3 sets X 10 reps daily) |
| 6 | SESSION 6  (POD 9) | - Review pleasant activity scheduling and practices - Introduce pleasant imagery - Safe and beneficial exercises, pace up of exercises - Spinal muscle strengthening - Range of motion exercises - Walking   Demonstration of home exercise program (Dose: 3 sets X 10 reps daily) |
| 7 | SESSION 7  (POD 14) | - Review pleasant imagery, distraction, and practices - Introduce concept of problem solving - Demonstrate problem solving - Strengthening exercises(Neutral spine control exercises with resistance) - Cardiovascular fitness: Walking or stationary cycling dose: 10-20 min - Integration of closed kinetic chain functional exercise - Demonstration of home exercise program ( Dose: 3 sets X 10 reps daily) |
| 8 | SESSION 8  (POD WEEK 4) | - Review content of all components - Reinforce body mechanics - Self Stretching - Integration of lumbopelvic stabilization exercise and more advanced closed kinetic chain function exercises - Open kinetic chain exercises - Cardiovascular fitness: Walking or stationary cycling dose: 20–30 min - Demonstration of home exercise program ( Dose: 3 sets X 10 reps daily) |

**APPENDIX: II**

Standard rehabilitation protocol for the control group

| **SL NO.** | **SESSION** | **CONTROL GROUP** |
| --- | --- | --- |
| 1 | SESSION 1  (POD 0) | - Patient education (Education on healing processes, physiological and psychological pain processes and Protection of incision) - Breathing exercises (abdominal lip-reduction breathing), 5–10 sets/time, 3 times/day. - DVT Prevention |
| 2 | SESSION 2  (POD 1) | - Patient education (Importance of safe exercise and rehabilitaion) - Isometric Muscle activation (Transversus abdominus in supine, isometric quadriceps, isometric gluteal and pelvic floor exercises) - Range of Motion exercise (Knee flection/extension, hip abduction/adduction, trunk side bends, ankle dorsi/plantarflexion) - Walking - Home exercise demonstration (Dose: 3 sets X 10 reps daily) |
| 3 | SESSION 3  (POD 3) | - Return to normal activity advises - Isometric Muscle activation (Transversus abdominus in supine, isometric quadriceps, isometric gluteal and pelvic floor exercises) - Range of motion exercises and Strengthening exercises   (Knee flection/extension, hip abduction/adduction, trunk side bends, ankle dorsi/plantarflexion, SLR, Pelvic bridging)   - Walking - Home exercise demonstration (Dose: 3 sets X 10 reps daily) |
| 4. | SESSION 4  (POD 5) | - Return to normal activity advises - Pace up of exercise - Isometric Muscle activation (Transversus abdominus in supine, isometric quadriceps, isometric gluteal and pelvic floor exercises) - Range of motion exercises and Strengthening exercises   (Knee flection/extension, hip abduction/adduction, trunk side bends, ankle dorsi/plantarflexion, SLR, Pelvic bridging)   - Home exercise demonstration (Dose: 3 sets X 10 reps daily)   (Progression of training intensity contingent to self-perceived pain.) |
| 5 | SESSION 5  (POD 7) | - Return to normal activity advises - Isometric Muscle activation (Transversus abdominus in supine, isometric quadriceps, isometric gluteal and pelvic floor exercises) - Range of motion exercises and Strengthening exercises   (Knee flection/extension, hip abduction/adduction, trunk side bends, ankle dorsi/plantarflexion, SLR, Pelvic bridging)   - Home exercise demonstration (Dose: 3 sets X 10 reps daily) |
| 6 | SESSION 6  (POD 9) | - Return to normal activity advises - Strengthening exercises (supine and prone SLR, Hip abduction and external rotation in side-lying position, Leg lift and knee extension with one leg in supine, hip abduction/adduction in side lying) - Range of motion exercise. - Walking   (Progression of training intensity contingent to self-perceived pain.)   - Home exercise demonstration (Dose: 3 sets X 10 reps daily) |
| 7 | SESSION 7  (POD 14) | - Patient Education (Importance of Safe and beneficial exercises) - Pace up of exercises - Strengthening (supine and prone SLR, Hip abduction and external rotation in side-lying position, Leg lift and knee extension with one leg in supine, hip abduction/adduction in side lying – with resistance) - Range of motion exercises - Home exercise demonstration (Dose: 3 sets X 10 reps daily) - Walking   (Progression of training intensity contingent to self-perceived pain.) |
| 8 | SESSION 8  (POD WEEK 4) | - Muscle strengthening and endurance exercises (supine buttock lifts, contralateral hip extension, and shoulder flexion in a 4-point kneeling position, prone push-ups) - Stretching exercises - Walking - Home exercise demonstration (Dose: 3 sets X 10 reps daily)   (Progression of training intensity contingent to self-perceived pain.) |

**APPENDIX: III**

**INFORMED CONSENT FORM**

**STUDY TITLE:**

Effect of Biopsychosocial Model of rehabilitation on Functional disability and Pain in patients after Robotic Assisted Lumbar fusion surgery: A Randomized Controlled Trial

**SUBJECT’S HOSPITAL NUMBER:** __________________

**SUBJECT’S NAME & AGE:** _______________________________

|  |  | SUBJECT INITIAL BOX |
| --- | --- | --- |
| 1 | The content of the above consent form and the procedure has been explained to me in a language_______ known to me and I have understood the same. |  |
| 2 | I understood that my participation in the study is voluntary and that I am free to withdraw any time, without my medical care or legal rights being affected. |  |
| 3 | I agree not to restrict the use of any data or results that arise from this study provided such a use is only for scientific purpose(s). |  |
| 4 | I agree to take part in the above study. |  |
| 5 | I have received a copy of the signed and dated informed consent form. |  |

**INFORMED CONSENT SIGNATURES**

**A)** Subject’s signature _______________ or Thumb impression

Date / Time ___________

Name of subject______________________________________

**B)** Witness’ signature ____________ or Thumb impression

Date / Time ___________

Name of Witness ______________________________________

**C)** Signature of guide _________________

Date / Time ____________

Name of the Investigator _____________

| 1. | SIGNATURE OF THE CANDIDATE: |  |
| --- | --- | --- |
| 2. | REMARKS OF THE GUIDE |  |
| 3. | 3.1 NAME AND DESIGNATION OF GUIDE  (in block letters) | DR. H. KARVANNAN  HEAD &ASSOCIATE PROFESSOR  DEPARTMENT OF PHYSIOTHERAPY  MCHP BANGALORE |
|  | 3.2 SIGNATURE |  |
|  | 3.3 CO GUIDE | Dr. Vidyadhara S  Head and Consultant  Manipal Comprehensive Spine Care Center  Manipal Institute of Robotic Spine Surgery  Manipal Hospitals Bangalore |
|  | 3.4 SIGNATURE |  |
|  | 3.5 HEAD OF THE DEPARTMENT | DR. H. KARVANNAN  HEAD &ASSOCIATE PROFESSOR  DEPARTMENT OF PHYSIOTHERAPY  MCHP BANGALORE |
|  | 3.6 SIGNATURE |  |
